# Chemoselective Covalent Modification of K-Ras(G12R) with a Small Molecule Electrophile

**DOI:** 10.1021/jacs.2c05377

**Published:** 2022-08-24

**Authors:** Ziyang Zhang, Johannes Morstein, Andrew K. Ecker, Keelan Z. Guiley, Kevan M. Shokat

**Affiliations:** † Department of Cellular and Molecular Pharmacology and Howard Hughes Medical Institute, University of California San Francisco, San Francisco, California 94158, United States; ‡ Department of Chemistry, University of California Berkeley, Berkeley, California 94720, United States

## Abstract

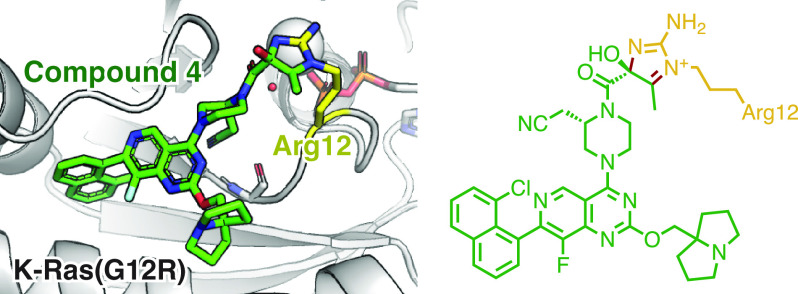

*KRAS* mutations are one of the most common oncogenic drivers in human cancer. While small molecule inhibitors for the G12C mutant have been successfully developed, allele-specific inhibition for other *KRAS* hotspot mutants remains challenging. Here we report the discovery of covalent chemical ligands for the common oncogenic mutant K-Ras(G12R). These ligands bind in the Switch II pocket and irreversibly react with the mutant arginine residue. An X-ray crystal structure reveals an imidazolium condensation product formed between the α,β-diketoamide ligand and the ε- and η-nitrogens of arginine 12. Our results show that arginine residues can be selectively targeted with small molecule electrophiles despite their weak nucleophilicity and provide the basis for the development of mutant-specific therapies for K-Ras(G12R)-driven cancer.

Somatic mutations of the *KRAS* proto-oncogene are the predominant oncogenic lesions in human cancer.^1,2^ Although *KRAS* was historically considered an “undruggable” target, the recent successful development of covalent K-Ras(G12C) ligands has demonstrated remarkable clinical benefits of direct, allele-specific K-Ras inhibition.^3,4^ These K-Ras(G12C) inhibitors exploit the strong nucleophilicity of the mutant cysteine and irreversibly bind in the Switch II region of K-Ras.^5−11^ However, many frequently occurring somatic mutations of K-Ras do not yield cysteine residues, and selective targeting of these mutants remains an unmet challenge. One of such hot spot mutations, *KRAS p.*G12R, is found in 17% of pancreatic ductal adenocarcinoma (PDAC) patients, accounting for more than 9,000 new cancer patients per year in the U.S. alone.^4,12^ We reasoned that covalent engagement of the acquired arginine (Arg12) could impart both potency and selectivity and enable the direct targeting of K-Ras(G12R).

Fully protonated at physiological pH (p*K*_a__3_ = 12.5), the guanidium group of arginine is weakly nucleophilic. To achieve chemoselective targeting of the mutant arginine, we first considered functional groups privileged to react with guanidines or amidines. Following the discovery of the reaction between 2,3-butanedione and benzamidine by Diels and Schleich in 1916,^13^ a variety of vicinal dicarbonyl compounds^14−20^ (e.g., phenylglyoxal,^14^ methylglyoxal,^20−22^ 2,3-butanedione,^15^ 1,2-cyclohexadionone^23^) have been found to selectively modify arginine residues on proteins. While these reagents have been employed to study protein glycation,^16,22,24−28^ nonspecifically modify proteins^23,29,30^ and perform bioconjugation reactions,^19,31−33^ such reactivity has not been exploited in the design of targeted covalent ligands.

We asked whether a K-Ras Switch-II ligand, when equipped with a vicinal dicarbonyl system, would react with Arg12 in K-Ras(G12R). We synthesized compound **3** (Figure 1A) by acetoacetylation of piperazine **1** followed by α-oxidation of the resulting acetoacetamide **2** with Dess–Martin periodinane.^34^ Compound **3** possesses an α,β-diketoamide, a rare but naturally occurring function present in FK506 and rapamycin. Likely due to the strong electrophilicity of the α-ketone, compound **3** could only be isolated as a hydrate. Compound **3** was stable in aqueous buffers over a range of pH and did not react with common thiol-containing nucleophiles (2-mercaptoethanol, dithiothreitol, see Figure S2 for details). However, when we incubated 100 μM compound **3** (molecular weight = 674 Da) with recombinant K-Ras(G12R)·GDP (GDP = guanosine diphosphate) at pH 7.5 at 23 °C and monitored the reaction by intact protein mass spectrometry, we observed the formation of two new protein species with molecular weights consistent with a stoichiometric covalent adduct with the loss of one or two equivalents of water (+656 Da and +638 Da, respectively, Figure 1C). Such reactivity is unique to the α,β-diketoamide **3**, as compound **2** did not form any detectable covalent adduct under the identical reaction conditions. We hypothesized that the products of this reaction included imidazoline adducts with Arg12 and their dehydration products (Figure 1B),^35^ although their chemical identity remained to be determined.

**Figure 1:**
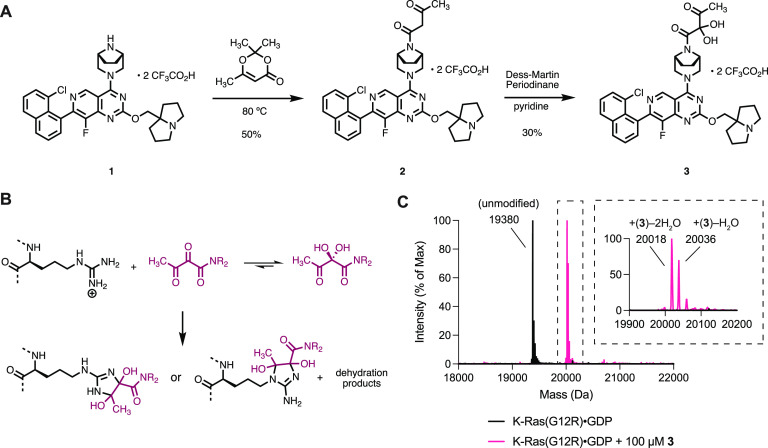
(A) Synthesis of α,β-diketoamide **3**. (B) Scheme depicting the reaction between an arginine residue and an α,β-diketoamide. (C) Intact protein mass spectra of K-Ras(G12R)·GDP and K-Ras(G12R)·GDP·**3** adduct.

Compound **3** did not modify wildtype (WT) K-Ras·GDP (Figure 2A) or other hotspot mutants including G12D, G12V, Q61R, Q61K and Q61L (Figure 2B) upon extended incubation at pH 7.5, confirming the specificity of its reaction with Arg12. Additionally, K-Ras contains several surface exposed arginines, which were not modified by compound **3**, further supporting its selectivity for the mutant arginine. Consistent with the preference of this ligand scaffold for the GDP-bound state of the protein, compound **3** reacted much slower with K-Ras(G12R)·GppNHp, with <5% modification after 72 h (Figure 2A). The reaction was pH-dependent and proceeded at greatly reduced rate at pH 6. The reaction rate reached maximum at pH 8 and did not further increase at pH 9 (Figure 2C). The K-Ras(G12R)·GDP·**3** adduct exhibited markedly increased thermostability compared to unmodified K-Ras(G12R)·GDP (Figure 2D), with an increase of melting temperature by 9.1 °C. The formation of this adduct also appeared irreversible: we did not observe any reversal to the unmodified protein after incubation of the purified K-Ras(G12R)·GDP·**3** adduct at pH 7.5 for 7 days at 23 °C.

**Figure 2:**
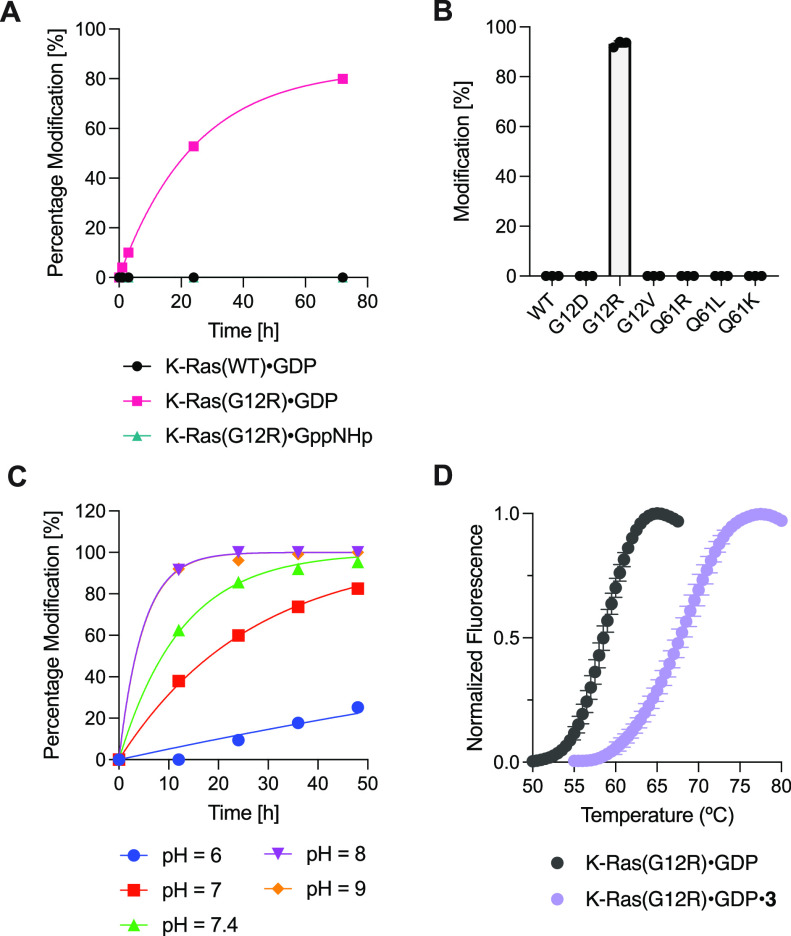
(A) Time-dependent covalent modification of wildtype K-Ras and CysLight K-Ras(G12R) by compound **3** (50 μM). (B) Reaction between K-Ras mutants and compound **3** (50 μM, 16 h). (C) Reaction between K-Ras(G12R)·GDP and compound **3** (50 μM) at various pH. (D) Differential scanning fluorimetry of K-Ras(G12R)·GDP and K-Ras(G12R)·GDP·**3** adduct.

To understand the chemical nature of the adduct formed between Arg12 and α,β-diketoamides, we obtained a cocrystal of K-Ras(G12R)·GDP and compound **4** (a structural analog of compound **3**, see Supporting Information Figure S3 for its biochemical characterization) in the space group P2_1_, which diffracted to 1.7 Å (Figure 3A). Compound **4** was bound in the Switch II pocket of K-Ras, with well-defined electron density for the covalent bonds between the ligand and the protein (Figure 3A). Surprisingly, we observed an imidazolium structure where Arg12 participated in a “side-on” orientation with its ε and η nitrogens. Such an imidazolium adduct is consistent with the loss of two water molecules revealed by whole protein mass spectrometry (Figure 1C). The nucleophilic addition from the η nitrogen also appeared to be stereoselective, as the electron density clearly indicates the tertiary alcohol to be of *S*- configuration (Figure 3A,B). Compared with unliganded K-Ras(G12R)·GDP, the side chain of Arg12 moved closer to the Switch II region, and the C_β_-C_γ_-C_δ_-N_ε_ dihedral angle shifted from *anti* to *gauche* (Figure 3C). These are likely energetically costly movements compensated by the reaction with the α,β-diketoamide and represent opportunities for future ligand optimization. The conformation of our adduct also differs from that seen with covalent K-Ras(G12C) ligands such as MRTX849 (Figure 3D): the adduct is formed further away from the protein surface, and the amide carbonyl in our structure did not participate in a hydrogen bond interaction with Lys16.

**Figure 3:**
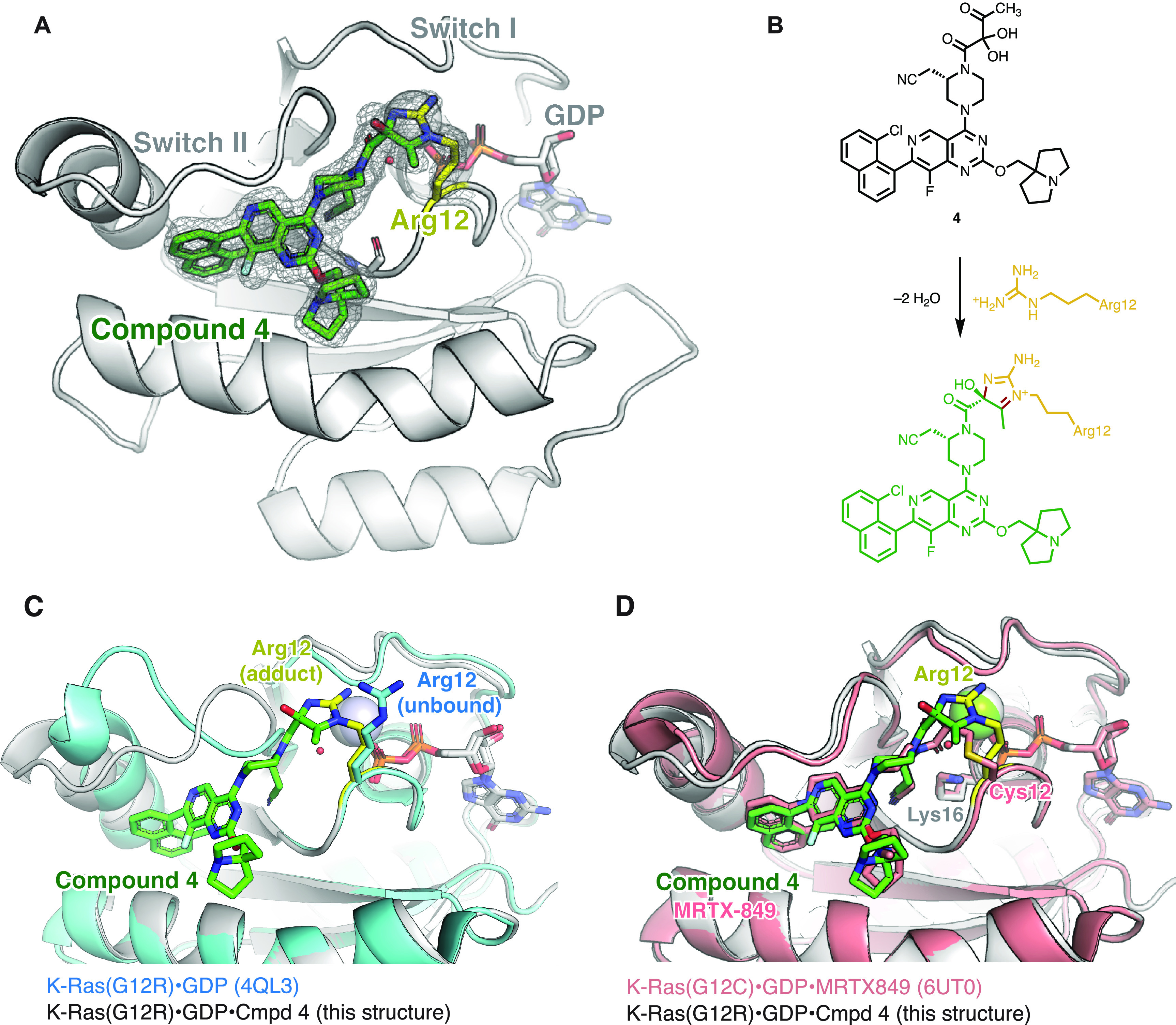
(A) Crystal structure of K-Ras(G12R)·GDP·**4** adduct. *F*_*o*_*–F*_*c*_ omit map is depicted for compound **4** and arginine 12 in gray mesh (σ = 2.0). (B) Scheme depicting the reaction between compound **4** and the Arg12 residue. (C) Comparison of the structures of unliganded K-Ras(G12R)·GDP (PDB: 4QL3) and K-Ras(G12R)·GDP·**4** adduct. (D) Comparison of the structures of K-Ras(G12C)·GDP·MRTX849 (PDB: 6UT0) and K-Ras(G12R)·GDP·**4** adduct.

Asking whether the reaction between **3** and Arg12 confers functional inhibition of K-Ras(G12R), we tested the nucleotide exchange activity of unliganded and compound **3**-bound K-Ras(G12R), where a fluorescent GDP analog (BODIPY-GDP) was exchanged for unlabeled GDP in the presence of son of sevenless (Sos) or ethylenediaminetetraacetic acid (EDTA) (Figure 4A). Compound **3** inhibited Sos-mediated exchange and significantly reduced the rate of EDTA-mediated exchange, consistent with previous observation with G12C-targeted covalent ligands.^5^

**Figure 4:**
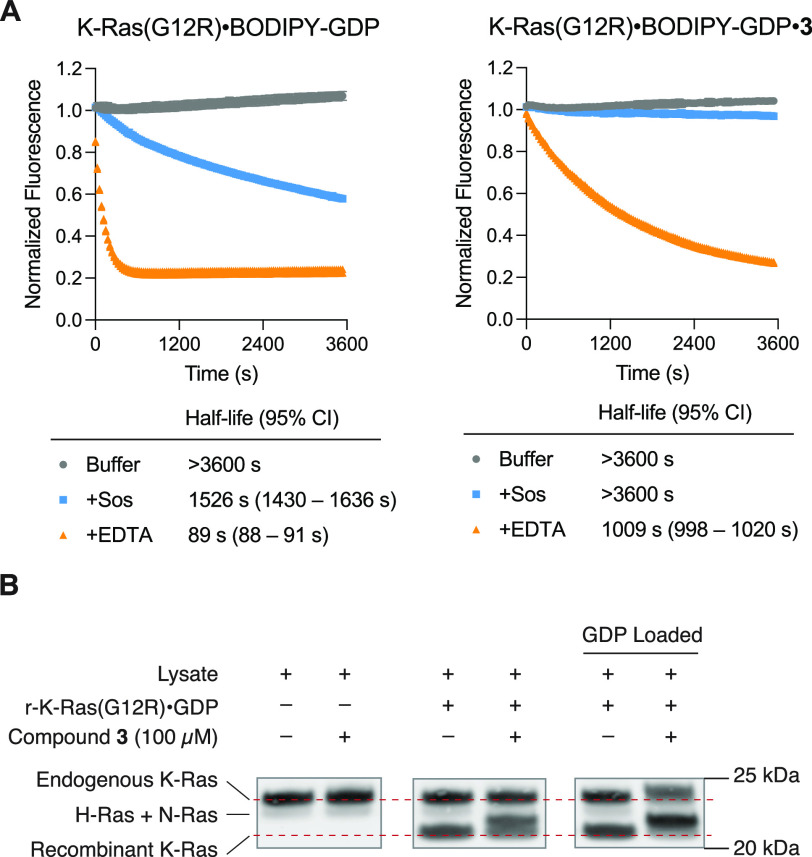
(A) Sos- or EDTA-mediated nucleotide exchange of K-Ras(G12R) and K-Ras(G12R)·**3** adduct. (B) Covalent modification of endogenous and exogenous K-Ras(G12R) in cell lysates.

To probe the covalent modification of K-Ras by our arginine-targeted electrophile in a complex proteome, we took advantage of the molecular weight increase upon the reaction between K-Ras and **3**, which is large enough to provide a direct visualization of target engagement on the anti-Ras immunoblot (Figure 4B). We treated the lysates of BaF3/K-Ras(G12R), a mouse cell line engineered to express the mutant form of K-Ras, with compound **3** and monitored the Ras molecular weight change. We did not detect any modification of the endogenous K-Ras(G12R). However, when we added recombinant K-Ras(G12R)·GDP to the lysate, we observed complete modification of this exogenous protein after 16 h of incubation (Figure 4B). The difference in reactivity suggested that compound **3** remained active in cell lysates but was unable to engage endogenous K-Ras(G12R). This was consistent with our observation that compounds **3** and **4** did not show cellular activity at concentrations below 100 μM (Supporting Information Figures S4 and S5). Unlike K-Ras(G12C), which has been successfully targeted by GDP-state selective ligands, K-Ras(G12R) is known to have severely compromised GTPase activity^12,36^ preventing its conversion into the susceptible GDP-bound state. We hypothesized that endogenous K-Ras(G12R) may exist predominantly in the GTP-bound state and therefore is not susceptible to **3** engagement. To test this hypothesis, we preloaded BaF3/K-Ras(G12R) lysates with excess GDP and repeated our treatments as above. Under these conditions, we observed covalent modification of the endogenous K-Ras(G12R), as evidenced by the upward shift of the Ras band (Figure 4B), confirming that the predominant GTP nucleotide state of K-Ras(G12R) can be a limiting factor for its therapeutic targeting. This represents an opportunity for future optimization–either by identifying ligand scaffolds that recognize K-Ras in the GTP-bound state, or by shifting K-Ras to its GDP-bound state with agents targeting upstream signaling nodes (e.g., RTK, SHP2 or Sos inhibitors).^37^

In conclusion, we have identified the first mutant-selective covalent ligands of K-Ras(G12R) using α,β-diketoamides as a privileged arginine-reactive functional group. We found that these ligands give rise to stable imidazolium adducts with K-Ras(G12R) and directly observed the structure of one such adduct using X-ray crystallography. While the reaction between vicinal dicarbonyl compounds and amidines/guanidines has been known since 1916, we show that this reactivity can be utilized in the design of targeted covalent ligands that engage weakly nucleophilic arginine residues in a complex proteome. An important limitation of our current work is that these unoptimized compounds do not show activity in KRAS G12R mutant cells. This is likely due to a combination of (1) suboptimal electrophile positioning of our compounds and (2) the preponderance of the GTP-bound form of K-Ras(G12R) in cells. However, our work presents an important first step in targeting this oncogene, and future medicinal chemistry optimization could yield more potent ligands that target the GTP-bound form. This has recently been shown with other mutants for K-Ras.^38,39^ Our discovery expands our ability to selectively target a recurrent oncogenic mutant, K-Ras(G12R), for which no direct inhibitors have been reported. The chemistry reported here may also serve as the basis for the therapeutic targeting of other acquired arginine residues in human diseases.

## Supporting Information Available

Supporting Information Figures S1–S6, Supporting Information Tables S1–S3, experimental procedures and characterization data for new compounds (PDF)

## Accession Codes

Atomic coordinates and structure factors for the crystal structure of K-Ras(G12R)·GDP·**4** adduct have been deposited with the Protein Data Bank (PDB) with the accession number 8CX5.
