# Supplementary material for: Chemoselective Covalent Modification of K-Ras(G12R) with a Small Molecule Electrophile
Source: J Am Chem Soc. 2022 Aug 24;144(35):15916–21. doi: 10.1021/jacs.2c05377 (PMC9460778; doi:10.1021/jacs.2c05377)
Supplement: Supplementary file 1 — ja2c05377_si_001.pdf [file ja2c05377_si_001.pdf]

Supporting Information for

Chemoselective covalent modification of K-Ras(G12R) with a small molecule electrophile

Ziyang Zhang[1,2,3*], Johannes Morstein[1,3], Andrew K. Ecker[1,3], Keelan Z. Guiley[1], and Kevan M. Shokat[1,2*]

[1] Department of Cellular and Molecular Pharmacology and Howard Hughes Medical Institute, University of California, San Francisco, California

[2] Department of Chemistry, University of California, Berkeley, California

[3] These authors contributed equally.

Table of Contents

Supplementary Figures and Tables 4

Figure S1. Stability of compound **3** at various pH. 4

Figure S2. Stability of compound **3** in the presence of thiol nucleophiles. 5

Figure S3. Biochemical characterization of compound **4**. 6

Figure S4. Cellular activity of compound **3** 7

Figure S5. Growth inhibition of BaF3/G12R cells (± IL-3) by compound **4**. 8

Figure S6. Permeability of Compound 3 assessed in a parallel artificial membrane permeability assay (PAMPA) 9

Table S1. X-ray crystallography data collection and refinement statistics 10

Table S2. List of antibodies 11

Table S3. List of buffer compositions 12

Safety notes 13

Cell culture 13

Lysate target engagement assay 13

Gel electrophoresis and immunoblot 13

Preparation of Mouse Stem Cell Virus (MSCV) 14

Generation of stable Ba/F3 transductants 14

Intact protein mass spectrometry 14

Sos- or EDTA-mediated nucleotide exchange assay 15

Differential scanning fluorimetry 15

Cell viability assay 15

Recombinant protein expression and purification 16

K-Ras (wildtype), K-Ras (G12R) and K-Ras (G12R) Cyslight 16

Sos^{cat} 16

Crystallization 17

X-Ray Data Collection and Structure Determination 17

Parallel Artificial Membrane Permeability Assay (PAMPA) 17

Chemical Synthesis 19

NMR Spectra 24

Supplementary Figures and Tables

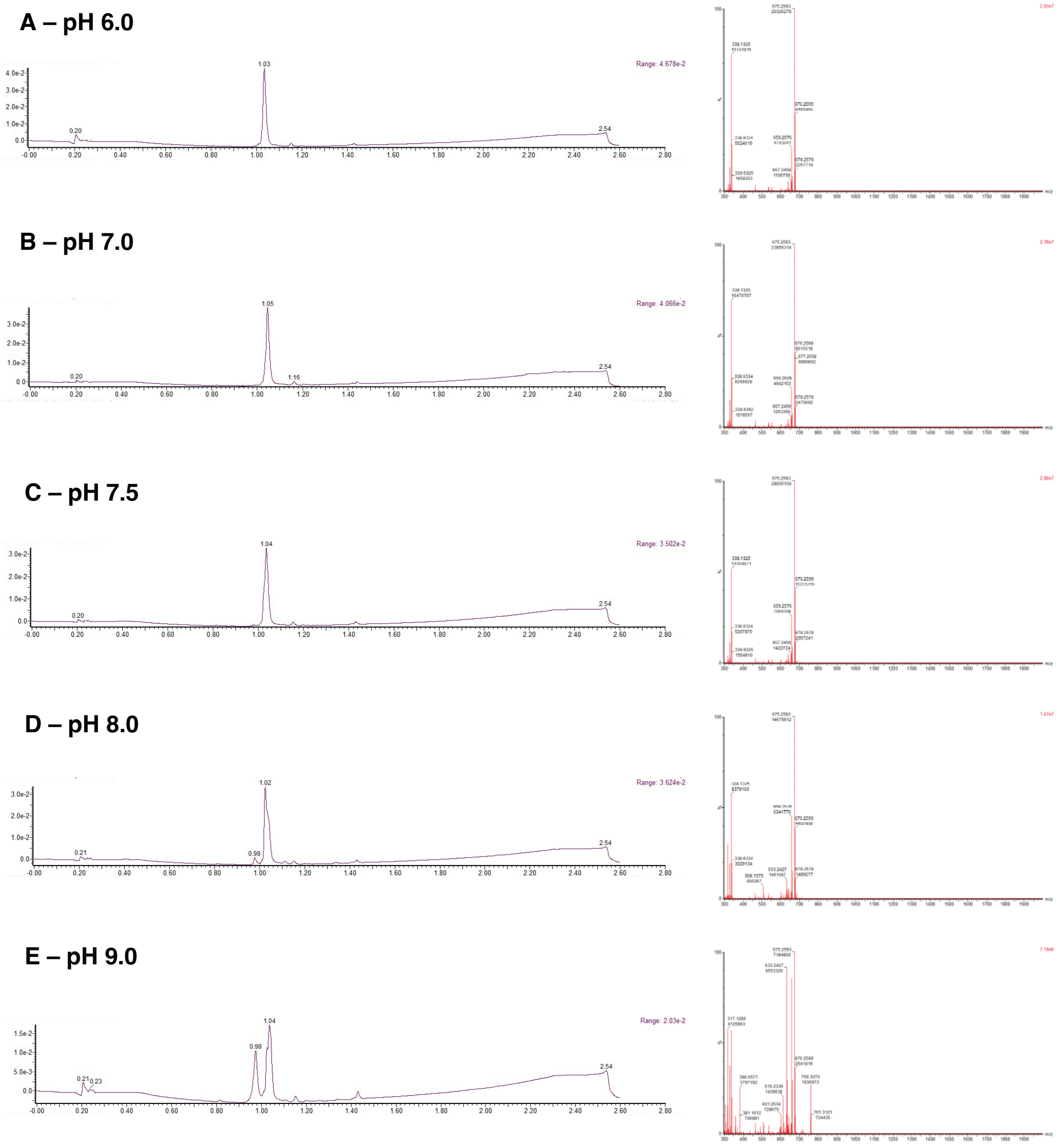

Figure S1. Stability of compound **3** at various pH. (A-E) Samples were analyzed by LC-MS after incubation in aqueous buffers at 23 ºC for 40 h. At pH>8.5 we started to see appreciable conversion of compound **3** to a new species with a loss of 42 Da in mass.

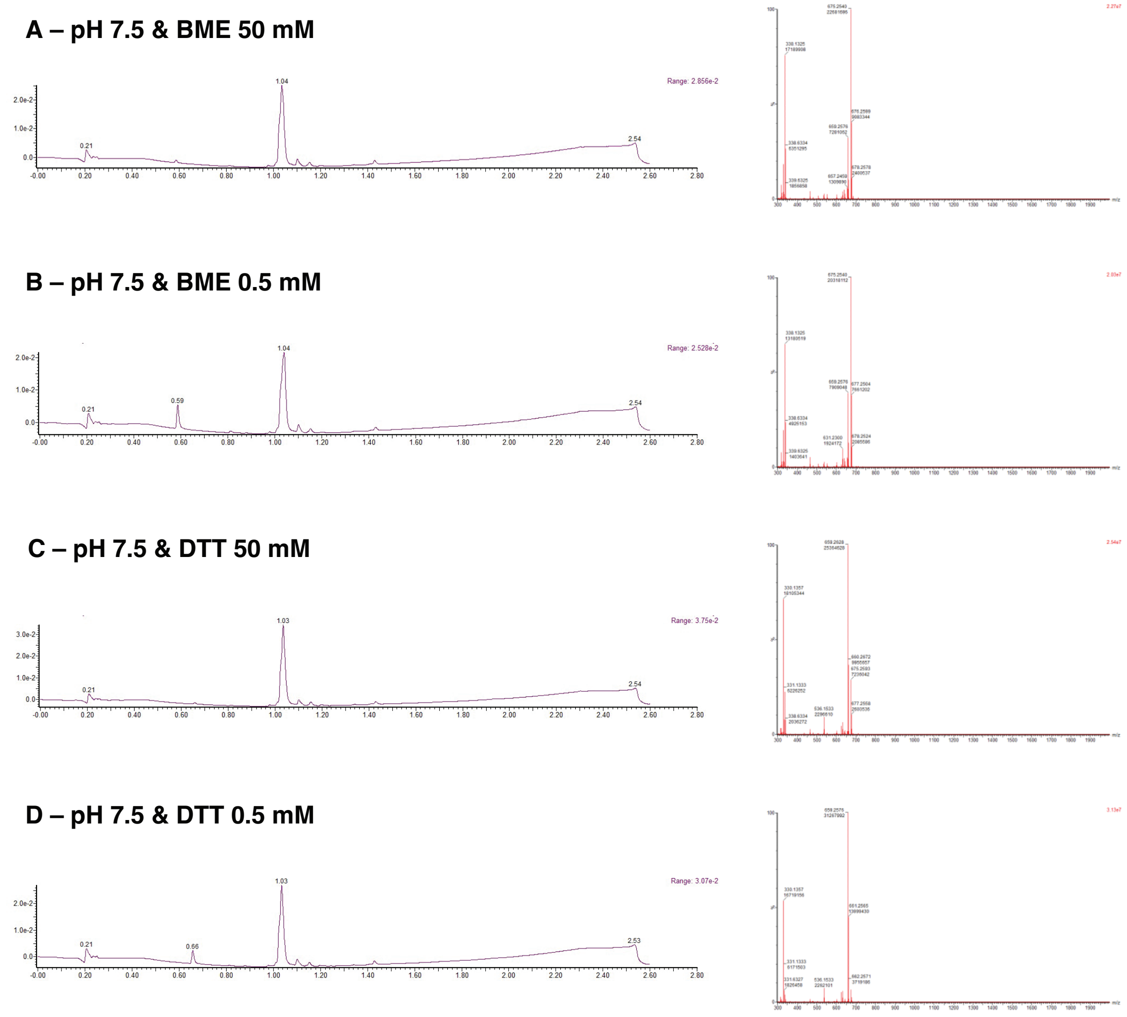

Figure S2. Stability of compound **3** in the presence of thiol nucleophiles. (A-D) Samples were analyzed by LC-MS after incubation in 20 mM HEPES 7.5, 150 mM NaCl, 1 mM $MgCl_2$ at 23 ºC for 40 h. We noticed that the ketone/hydrate ratio of compound **3** had shifted to favor the keto-form in DTT-treated samples (C-D).

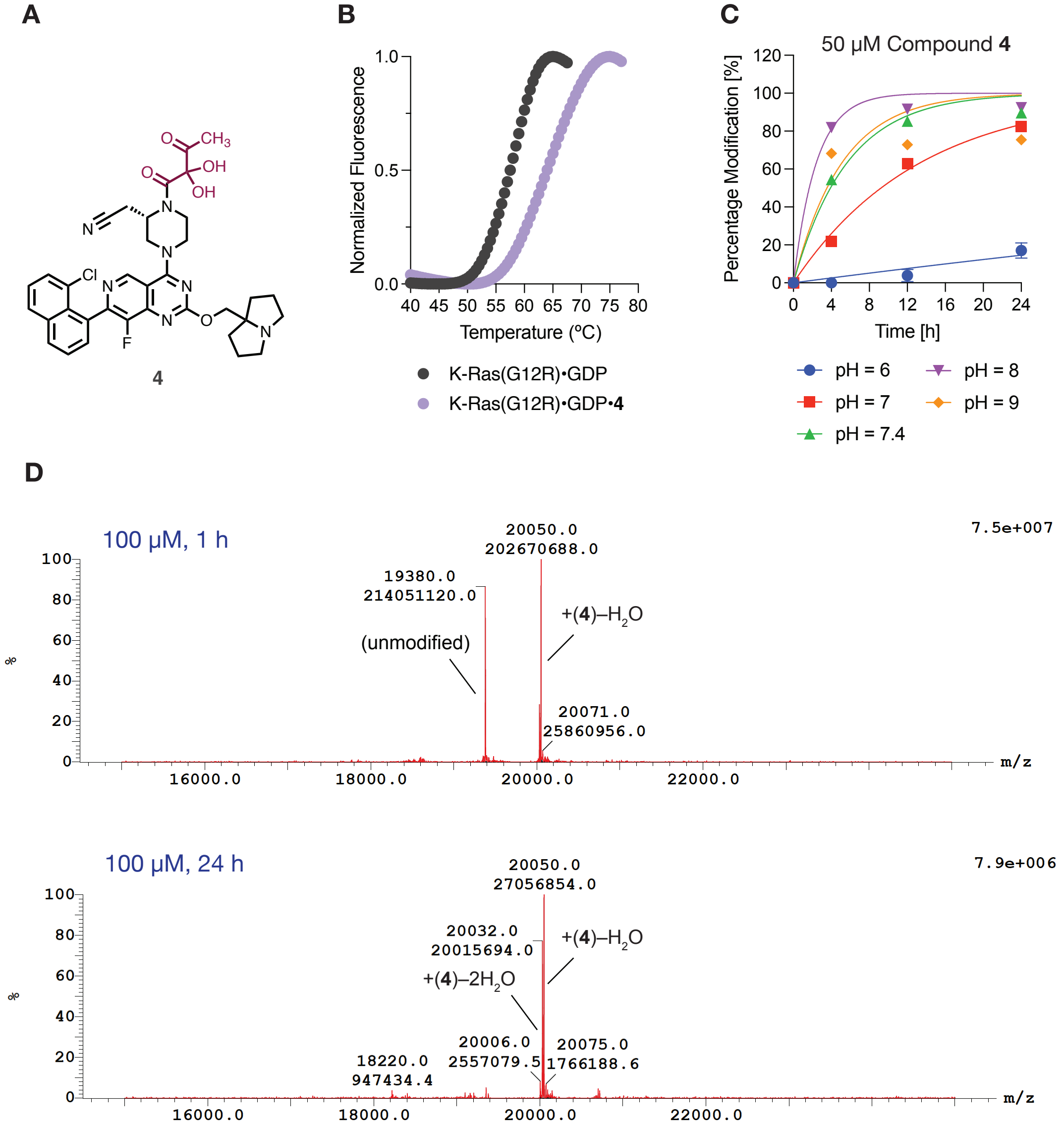

Figure S3. Biochemical characterization of compound **4**. (A) Structure of compound **4**. (B) Differential scanning fluorimetry of K-Ras(G12R)•GDP and its adduct with compound **4**. (C) Reaction between K-Ras(G12R)•GDP and compound **4** (50 μM) at various pH. (D) Whole protein mass spectrometry of K-Ras(G12R)•GDP incubated with 100 μM **4** at pH 7.5 for 1 h and for 24 h.

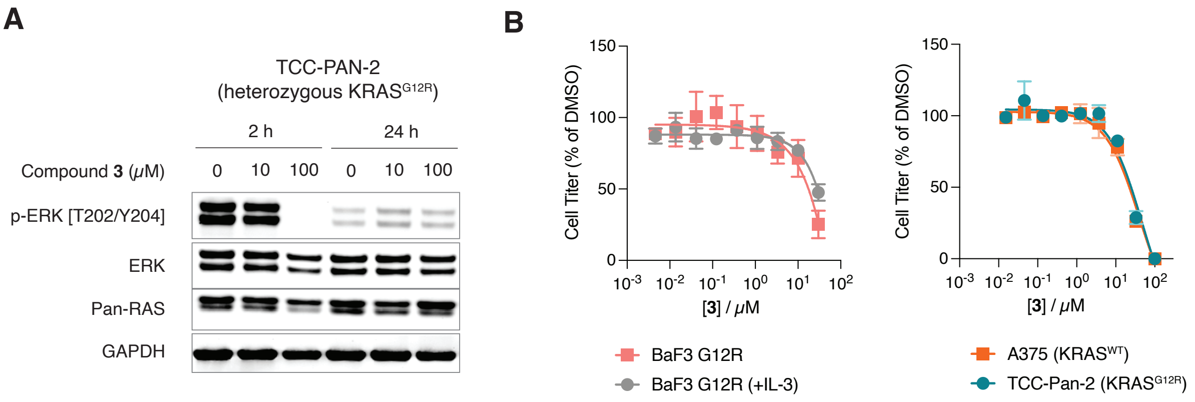

Figure S4. Cellular activity of compound **3**. A) Immunoblot analysis of phospho-ERK signaling in TCC-PAN-2 cells treated with compound 3. B) Growth inhibition of BaF3/G12R cells (± IL-3), TCC-Pan-2 cells, and A375 cells by compound 3.

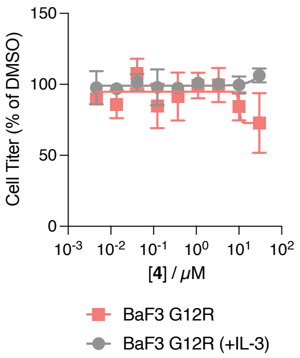

Figure S5. Growth inhibition of BaF3/G12R cells (± IL-3) by compound **4**.

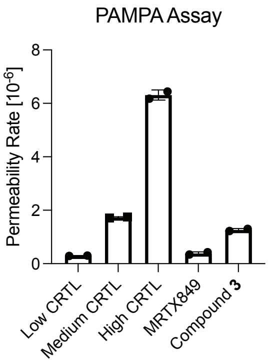

Figure S6. Permeability of compound **3** assessed in a parallel artificial membrane permeability assay (PAMPA). Three permeability controls were included: Chloramphenicol (high), Diclofenac (medium), and Theophylline (low). MRTX849 was included as a reference compound.

Table S1. X-ray crystallography data collection and refinement statistics

Values in parentheses are for highest resolution shell.

	K-Ras(G12R)•Compound **4**
Data collection	
Space group	$P12_11$
Cell dimensions	
a, *b*, *c* (Å)	41.21, 65.66, 61.13
α, β, γ (°)	90, 101.85, 90
Resolution (Å)	65.66-1.72 (1.75-1.72)
R_{merge}	0.156 (1.879)
R_{pim}	0.071 (0.992)
$I / \sigma I$	10.7 (1.7)
CC 1/2	0.974 (0.417)
Total reflections	191006 (7253)
Unique Reflections	32559 (1640)
Completeness (%)	96.3 (92.8)
Redundancy	5.9 (4.6)
Refinement	
Resolution (Å)	59.83-1.72 (1.78-1.72)
No. reflections	32312 (3089)
R_{work} / R_{free}	17.9/21.9
No. atoms	3284
Protein	2800
Ligands	119
Refined B-factors (Å^2)	
Overall	24.86
Compound	23.36
water	28.47
R.m.s. deviations	
Bond lengths (Å)	0.022
Bond angles (°)	1.65
Ramachandran analysis	
Favored (%)	96
Disallowed (%)	0.3

Table S2. List of antibodies

Target	Supplier	Identifier	Dilution
P-AKT [S473]	Cell Signaling Technology	4060	1:1000
P-AKT [T308]	Cell Signaling Technology	4056	1:1000
AKT	Cell Signaling Technology	2920	1:1000
P-ERK [T202/Y204]	Cell Signaling Technology	9101	1:1000
Total ERK	Cell Signaling Technology	4695	1:1000
Pan-Ras	Abcam	108602	1:5000
GAPDH	Proteintech	60004-1-Ig	1:50000

Table S3. List of buffer compositions

Name	Composition
RIPA Buffer	25 mM Tris 7.4<br>150 mM NaCl<br>0.1% SDS<br>1% NP-40<br>0.5% sodium deoxycholate
NP40 Buffer	100 mM HEPES 7.5<br>150 mM NaCl<br>0.1% NP-40<br>Protease Inhibitor
5x SDS Loading Buffer	250 mM Tris 6.8<br>500 mM DTT<br>10% w/v SDS<br>0.1% w/v Bromophenol Blue<br>50% Glycerol
TOWBIN Buffer	25 mM Tris<br>192 mM Glycine<br>pH 8.3
Lysis Buffer	20 mM Tris 8.0<br>500 mM NaCl<br>5 mM imidazole
Elution Buffer	20 mM Tris 8.0<br>300 mM NaCl<br>300 mM imidazole
TEV Cleavage Buffer	20 mM Tris 8.0<br>300 mM NaCl<br>1 mM EDTA<br>1 mM DTT
Phosphatase Buffer	32 mM Tris 8.0<br>200 mM ammonium sulfate<br>0.1 mM $ZnCl_2$
SEC Buffer	20 mM HEPES 7.5<br>150 mM NaCl<br>1 mM $MgCl_2$
Nucleotide Exchange Buffer	20 mM HEPES 7.5<br>150 mM NaCl<br>1 mM $MgCl_2$<br>1 mM DTT

Safety notes

All experiments were performed with standard personal protective equipment (PPE). All chemical syntheses were performed in ventilated fume hoods operating at a face velocity greater than 90 fpm. Handling of Biosafety Level 2 materials was performed according to UCSF Office of Environment, Health and Safety standards. No unexpected or unusually high safety hazards were encountered.

Cell culture

Ba/F3 cells were a gift from Dr. Trevor Bivona (UCSF) and were maintained in RPMI 1640 (Gibco 11875093) supplemented with 10% heat-inactivated fetal bovine serum (Axenia Biologix) and 10 ng/mL recombinant mouse interleukin-3 (Gibco PMC0031). Cells were passed for at least two generations after cryorecovery before they were used for assays. All cell lines were tested mycoplasma negative using MycoAlert™ Mycoplasma Detection Kit (Lonza).

When indicated, cells were treated with drugs at 40-60% confluency at a final DMSO concentration of 1%. At the end of treatment period, cells were placed on ice. Unless otherwise indicated, adherent cells were washed once with ice-cold PBS (1 mL), scraped with a spatula, and pelleted by centrifugation (500 x g, 5 min). Suspension cells were pelleted by centrifugation (500 x g, 5 min), washed with 1 mL ice-cold PBS, and pelleted again. Cells were lysed in RIPA buffer supplemented with protease and phosphatase inhibitors (cOmplete and phosSTOP, Roche) on ice for 10 min. Lysates were clarified by high-speed centrifugation (19,000 x g, 10 min). Concentrations of lysates were determined with protein BCA assay (Thermo Fisher) and adjusted to 2 mg/mL with additional RIPA buffer (or Co-IP Lysis Buffer). Samples were mixed with 5x SDS Loading Dye and heated at 95 ºC for 5 min.

Lysate target engagement assay

For co-treatment with recombinant K-Ras(G12R), Ba/F3(G12R) cells were lysed with NP-40 buffer and adjusted to 1.5 mg/ml. A 0.1 μM solution of recombinant K-Ras(G12R) was prepared in NP-40 buffer. 18μL of Ba/F3 lysate were added to 4 μL of a 0.5 mM solution of **3** containing 10% DMSO in NP-40 buffer or DMSO as control. 18μL of recombinant K-Ras(G12R) solution or NP-40 was added and the mixtures were incubated for 16h at 23 ºC. Subsequently samples were mixed with 5x SDS Loading Dye and heated at 95 ºC for 5 min.

Gel electrophoresis and immunoblot

Unless otherwise noted, SDS-PAGE was run with Novex 12% Bis-Tris gel (Invitrogen) in MES running buffer (Invitrogen) at 200 V for 60 min following the manufacturer's instructions. Protein bands were transferred onto 0.2-µm nitrocellulose membranes (Bio-Rad) using a wet-tank transfer apparatus (Bio-Rad Criterion Blotter) in 1x TOWBIN buffer with 10% methanol at 75V for 45 min. Membranes were blocked in 5% BSA–TBST for 1 h at 23 ºC. Primary antibody binding was performed with the indicated antibodies diluted in 5% BSA–TBST at 4 ºC for at least 16 h. After washing the membrane three times with TBST (5 min each wash), secondary antibodies (goat anti-rabbit IgG-IRDye 800 and goat anti-mouse IgG-IRDye 680, Li-COR) were added as

solutions in 5% skim milk–TBST at the dilutions recommended by the manufacturer. Secondary antibody binding was allowed to proceed for 1 h at 23 ºC. The membrane was washed three times with TBST (5 min each wash) and imaged on a Li-COR Odyssey fluorescence imager.

Preparation of Mouse Stem Cell Virus (MSCV)

pMSCV-Puro plasmids containing full length human *KRAS* genes (G12R) were constructed using standard molecule biology techniques by inserting the *KRAS* gene fragment between the BamHI and XhoI sites. Transfection-grade plasmids were prepared using ZymoPure II Plasmid Midiprep kit. EcoPack 293 cells (Takara Bio) were plated in 6-well plates (3×10^5/mL, 2 mL). The next day, cells were transfected with 2.5 µg pMSCV plasmid using lipofectamine 3000 following the manufacturer's instructions. The cells were incubated for 66 h, and then the virus-containing supernatants were collected and passed through a 0.22-µm syringe filter. The harvested virus was used immediately for spinfection of Ba/F3 cells or stored at –80 ºC.

Generation of stable Ba/F3 transductants

1 mL of MSCV-containing supernatant (*vide supra*) was added to one well of a 6-well plate containing 1×10^6 Ba/F3 cells in 1 mL of media comprised of 60% RMPI 1640, 40% heat-inactivated FBS, 10 ng mouse IL-3 and 4 µg polybrene. Cells were spinfected by centrifugation at 2,000 g for 90 minutes at room temperature and then placed in the incubator for 24 hours. After 1 day, the cells were diluted into 10 mL culture medium (RPMI 1640 + 10% heat-inactivated FBS, 10 ng/mL mouse IL-3) and recovered for a second day after spinfection. On the third day after spinfection, cells were pelleted at 500 x g for 5 min and resuspended in 10 mL selection medium (RPMI 1640 + 10% heat-inactivated FBS, 10 ng/mL mouse IL-3, 1.25 µg/mL puromycin). Cells were maintained under puromycin selection for 4-7 days, splitting as required to maintain density $<2 \times 10^6$ cells/mL. After 7 days, cells were pelleted, washed once with IL-3 free culture medium (RPMI 1640 + 10% heat-inactivated FBS) and pelleted again before resuspending at $2\text{-}4 \times 10^5$ cells/mL in IL-3 free culture medium. Cells were maintained under these conditions for 7 days, passaging as needed to maintain density $< 2 \times 10^6$ cells/mL. Growth was monitored (Countess II Cell Counter) over these 7 days to confirm that an IL-3 independent population has been achieved.

Intact protein mass spectrometry

Purified K-Ras variants (4 µM final) were incubated with compounds at 50 or 100 µM (1% v/v DMSO final) in 20 mM HEPES pH 7.5, 150 mM NaCl, 1 mM $MgCl_2$ in a total volume of 150 µL. After the noted time, the samples were analyzed by intact protein LC/MS using a Waters Xevo G2-XS system equipped with an Acquity UPLC BEH C4 1.7 µm column. The mobile phase was a linear gradient of 5–95% acetonitrile / water + 0.05% formic acid. The spectra were processed using QuantLynx, giving the ion counts observed for the most abundant species. For assays conducted under an alternate pH, the HEPES buffer was replaced with one of the following buffers (maintaining 150 mM NaCl and 1 mM MgCl2): 20 mM MES pH 6 or 20 mM Tris pH 9.

Sos- or EDTA-mediated nucleotide exchange assay

This assay was performed as previously reported[1–4] with slight modifications. BODIPY-GDP-loaded K-Ras proteins were prepared freshly as follows. To a 10 µM solution of K-Ras(G12R)•GDP or K-Ras(G12R)•GDP•**3** in SEC Buffer (1 mL) was added sequentially BODIPY-GDP (5 mM, 40 µL, Thermo Fisher, final concentration 200 µM) and Na-EDTA pH 8.0 (0.5 M, 5 µL, final concentration 2.5 mM). The mixture was incubated at 23 ºC for 1 h, and a solution of $MgCl_2$ (1.0 M, 20 µL, final concentration 10 mM) was added to the reaction mixture. The protein solution was run through a PD-10 column to remove the excess nucleotide following the manufacturer's protocol. Briefly, sample (~1.0 mL) and excess buffer (1.5 mL) were loaded onto the column (equilibrated with NucEx Buffer), and desalted protein was eluted with NucEx Buffer (3.5 mL). Desalted protein was diluted 1:1 to a concentration of roughly 1.5 µM in NucEx Buffer. 12 µL of this solution (triplicate for each condition) was added to wells of a black 384-well low-volume assay plate (Corning 4514). 3 µL of either 1 mM GDP, 1 mM GDP + 5 µM Sos, or 1 mM GDP + 40 mM EDTA (all prepared in NucEx Buffer) was added via a multichannel pipet rapidly to the wells. This should take less than 15 s to finish. The plate was immediately placed in a TECAN Spark 20M plate reader, and fluorescence for BODIPY (excitation 488 nm, emission 520 nm) was read every 30 s over 1 h. Fluorescence intensity was normalized to values at time 0 and plotted again time. Observed rate constant (k_{obs}) was derived by fitting the curve to first-order kinetic equation

$$F = (F_0 - F_\infty) \exp[-k_{obs}t] + F_\infty$$

and plotted against time.

Differential scanning fluorimetry

The protein of interest was diluted with SEC Buffer [20 mM HEPES 7.5, 150 mM NaCl, 1 mM $MgCl_2$] to 8 µM. This solution was dispensed into wells of a white 96-well PCR plate in triplicate (25 µL/well). Fluorescence was measured at 0.5-ºC temperature intervals every 30 s from 25 ºC to 95 ºC on a Bio-Rad CFX96 qPCR system using the FRET setting. Each data set was normalized to the highest fluorescence and the normalized fluorescence reading was plotted against temperature in GraphPad Prism 8.0. Tm values were determined as the temperature(s) corresponding to the maximum(ma) of the first derivative of the curve.

Cell viability assay

Cells were seeded into 96-well white flat bottom plates (1,000 cells/well) (Corning) and incubated overnight. Cells were treated with the indicated compounds in a nine-point threefold dilution series (100 µL final volume) and incubated for 72 h. Cell viability was assessed using a commercial CellTiter-Glo (CTG) luminescence-based assay (Promega). Briefly, the 96-well plates were equilibrated to room temperature before the addition of diluted CTG reagent (100 µL) (1:4 CTG reagent:PBS). Plates were placed on an orbital shaker for 30 min before recording luminescence using a Spark 20M (Tecan) plate reader.

Recombinant protein expression and purification

K-Ras (wildtype), K-Ras (G12R) and K-Ras (G12R) Cyslight

DNA sequences encoding human K-Ras (wildtype, aa 1-169), human K-Ras (G12R, aa 1-169), human K-Ras G12R Cyslight (G12R/C51S/C80L/C118S, aa 1-169), human NF1-GRD (aa 1203-1530) were codon optimized, synthesized by Twist Biosciences and cloned into pJExpress411 vector using the Gibson Assembly method[5]. The resulting construct contains N-terminal 6xHis tag and a TEV cleavage site (ENLYFQG). The proteins were expressed and purified following previously reported protocols.[1,6] Briefly, chemically competent BL21(DE3) cells were transformed with the corresponding plasmid and grown on LB agar plates containing 50 μg/mL kanamycin. A single colony was used to inoculate a culture at 37 ºC, 220 rpm in terrific broth containing 50 μg/mL kanamycin. When the optical density reached 0.6, the culture temperature was reduced to 20 ºC, and protein expression was induced by the addition of IPTG to 1 mM. After 16 h at 20 ºC, the cells were pelleted by centrifugation (6,500 x g, 10 min) and lysed in lysis buffer [20 mM Tris 8.0, 500 mM NaCl, 5 mM imidazole] with a high-pressure homogenizer (Microfluidics, Westwood, MA). The lysate was clarified by high-speed centrifugation (19,000 x g, 15 min) and the supernatant was used in subsequent purification by immobilized metal affinity chromatography (IMAC). His-TEV tagged protein was captured with Co-TALON resin (Clonetech, Takara Bio USA, 2 mL slurry/liter culture) at 4 ºC for 1 h with constant end-to-end mixing. The loaded beads were then washed with lysis buffer (50 mL/liter culture) and the protein was eluted with elution buffer [20 mM Tris 8.0, 300 mM NaCl, 300 mM imidazole]. To this protein solution was added His-tagged TEV protease (0.05 mg TEV/mg Ras protein) and GDP (1 mg/mg Ras protein), and the mixture was dialyzed against TEV Cleavage Buffer [20 mM Tris 8.0, 300 mM NaCl, 1 mM EDTA, 1 mM DTT] at 4 ºC using a 10K MWCO dialysis cassette until LC-MS analysis showed full cleavage (typically 16-24 h). $MgCl_2$ was added to a final concentration of 5 mM, and the mixture was incubated with 1 mL Ni-NTA (Qiagen) beads at 4 ºC for 1 h to remove TEV protease, any residual His-tagged proteins and peptides. The protein solution was diluted 1:10 v/v with 20 mM Tris 8.0 and further purified with anion exchange chromatography (HiTrapQ column, GE Healthcare Life Sciences) using a NaCl gradient of 50 mM to 500 mM in 20 mM Tris 8.0. Nucleotide loading was performed by mixing the ion exchange-purified protein with an excess of GDP (5 mg/liter culture) or GppNHp (5 mg/liter culture) and 5 mM EDTA at 23 ºC for 30 min. The reaction was stopped by the addition of $MgCl_2$ to 10 mM. For GppNHp, an additional calf intestine phosphatase treatment was performed as follows to ensure high homogeneity of the loaded nucleotide. The protein buffer was exchanged into Phosphatase Buffer [32 mM Tris 8.0, 200 mM ammonium sulfate, 0.1 mM $ZnCl_2$] with a HiTrap Desalting Column (GE Healthcare Life Sciences). To the buffer-exchanged protein solutions, GppNHp was added to 5 mg/mL, and Calf Intestine Phosphatase (NEB) was added to 10 U/mL. The reaction mixture was incubated on ice for 1 h, and $MgCl_2$ was added to a final concentration of 20 mM. After nucleotide loading, the protein was concentrated using an 10K MWCO centrifugal concentrator (Amicon-15, Millipore) to 20 mg/mL and purified by size exclusion chromatography on a Superdex 75 10/300 GL column (GE Healthcare Life Sciences). Fractions containing pure biotinylated Ras protein were pooled and concentrated to 20 mg/mL and stored at –78 ºC. In our hands, this protocol gives a typical yield of 5-15 mg/liter culture.

Sos^{cat}

The catalytic domain of Sos (residues 466-1049, Sos^{cat}) was expressed and purified following a published protocol.[7]

Crystallization

K-Ras(G12R) Cyslight (G12S/C51S/C80L/C118S) bound by GDP purified by size exclusion chromatography was diluted to 100 μM in Reaction Buffer (20 mM HEPES 7.5, 150 mM NaCl, 1 mM $MgCl_2$). Compound **4** was added as a 10 mM solution in DMSO to a final concentration of 200 μM. The mixture was allowed to stand at 23 ºC until LC-MS analysis of the reaction mixture showed full conversion to a single covalent adduct. The reaction mixture was purified by size exclusion chromatography (Superdex75, 20 mM HEPES 7.5, 150 mM NaCl, 1 mM $MgCl_2$) and concentrated to 20 mg/mL. For crystallization, 0.1 μL of the protein was mixed with 0.1 μL well buffer containing 0.1 M MES pH 6.5, 30% w/v PEG 4K. Crystals were grown at 20 ºC in a 96-well plate using the hanging-drop vapor diffusion method. Maximal crystal growth was achieved after 7 days. The crystals were transferred to a cryoprotectant solution (0.1 M MES pH 6.5, 30% w/v PEG 4K, 15% glycerol) and flash-frozen in liquid nitrogen.

X-Ray Data Collection and Structure Determination

Dataset was collected at the Advanced Light Source beamline 8.2.1 with X-ray at a wavelength of 0.999907 Å. The dataset was indexed and integrated using iMosflm (Battye et al., 2011), scaled with Scala (Evans, 2006) and solved by molecular replacement using Phaser (McCoy et al., 2007) in CCP4 software suite (Winn et al., 2011). The crystal structure of GDP-bound K-Ras(G12C)-MRTX849 adduct (PDB code: 6UT0) was used as the initial model. The structure was manually refined with Coot (Emsley et al., 2010) and PHENIX (Adams et al., 2010). Data collection and refinement statistics are listed in Table S1. In the Ramachandran plot of the final structure, 96.13% and 3.87% of the residues are in the favored regions and allowed regions, respectively.

Parallel Artificial Membrane Permeability Assay (PAMPA)

Parallel Artificial Membrane Permeability Assay was performed by BioAssay Systems Inc. (Hayward, CA). A 25 μL aliquot of each test compound was diluted into 475 μL PBS (pH 7.4). A 84 μL aliquot of the diluted compound was further diluted with 126 μL of PBS (pH 7.4) to serve as the Equilibrium Standard. A 4% lecithin lipid mixture was solubilized in 100% dodecane then 5 μL of the lecithin/dodecane solution was applied to the PAMPA Donor plate membrane. In duplicate, 200 μL of the initial compound dilution was applied to the PAMPA plate Donor well. A 300 μL aliquot of PBS (pH 7.4) was added to each Acceptor well then the PAMPA system was assembled. The PAMPA plate was incubated for 18 hours at room temperature. Following the incubation, 100 μL of each Acceptor solution was transferred to a 96-well UV plate. In parallel, each compound's Equilibrium solution was also added to the plate, in duplicate. If an Equilibrium solution showed precipitate, it was clarified by centrifugation at 13,000xg for 10 minutes. The Blank was DMSO in PBS (pH 7.4) minus compound at the same final dilution (2 %(v/v)). As a positive control, Chloramphenicol (high), Diclofenac (medium), and Theophylline (low) were run in parallel at 500 μM for the Donor solution and 200 μM for the Equilibrium solution. The permeability rate of each compound was determined using the following equation:

$$P_e = C \times -\ln\left(1 - \frac{OD_A}{OD_E}\right) cm/s$$

where Pe is the Permeability Rate, ODA is the absorbance of the Acceptor solution minus the Blank absorbance, ODE is the absorbance of the Equilibrium solution minus the Blank absorbance, and C = 7.72 $\times 10^{-6}$. The Peak of absorbance used for each compound was compound dependent and based upon peak of maximal absorbance in the Equilibrium solution.

Chemical Synthesis

General Experiment Procedure

All reactions were performed in oven-dried glassware fitted with rubber septa under a positive pressure of argon, unless otherwise noted. Air- and moisture-sensitive liquids were transferred via syringe. Solutions were concentrated by rotary evaporation at or below 40 °C. Analytical thin-layer chromatography (TLC) was performed using glass plates pre-coated with silica gel (0.25-mm, 60-Å pore size, 230−400 mesh, Merck KGA) impregnated with a fluorescent indicator (254 nm). TLC plates were visualized by exposure to ultraviolet light (UV), then were stained by submersion in a 10% solution of phosphomolybdic acid (PMA) in ethanol or an 2% aqueous solution of potassium permanganate followed by brief heating on a hot plate. Flash column chromatography was performed with Teledyne ISCO CombiFlash EZ Prep chromatography system, employing pre-packed silica gel cartridges (Teledyne ISCO RediSep).

Solvents and Reagents

Anhydrous solvents were purchased from Acros Organics. Unless specified below, all chemical reagents were purchased from Sigma-Aldrich, AK Scientific or Chemscene.

Instrumentation

Proton nuclear magnetic resonance (^{1}H NMR) spectra, carbon nuclear magnetic resonance (^{13}C NMR) spectra, and fluorine nuclear magnetic resonance (^{19}F NMR) spectra were recorded on Bruker AvanceIII HD instrument (400 MHz/100 MHz/376 MHz) at 23 °C operating with the Bruker Topspin 3.1. NMR spectra were processed using Mestrenova (version 14.1.2). Proton chemical shifts are expressed in parts per million (ppm, δ scale) and are referenced to residual protium in the NMR solvent ($CHCl_3$: δ 7.26, D_2HCOD: δ 3.31). Carbon chemical shifts are expressed in parts per million (ppm, δ scale) and are referenced to the carbon resonance of the NMR solvent ($CDCl_3$: δ 77.0, CD_3OD: δ 49.0). Fluorine chemical shifts are expressed in parts per million (ppm, δ scale) and are referenced to an external standard of trifluoroacetic acid (–76.55 ppm). Data are represented as follows: chemical shift, multiplicity (s = singlet, d = doublet, t = triplet, q = quartet, dd = doublet of doublets, dt = doublet of triplets, m = multiplet, br = broad, app = apparent), integration, and coupling constant (*J*) in Hertz (Hz). High-resolution mass spectra were obtained using a Waters Xevo G2-XS time-of-flight mass spectrometer operating with Waters MassLynx software (version 4.2).

Monitoring Reaction Progress by LC-MS

When LC-MS analysis of the reaction mixture is indicated in the procedure, it was performed as follows. An aliquot (1 µL) of the reaction mixture (or the organic phase of a mini-workup mixture) was diluted with 100 µL 1:1 acetonitrile:water. 1 µL of the diluted solution was injected onto a Waters Acquity UPLC BEH C18 1.7 µm column and eluted with a linear gradient of 5–95% acetonitrile/water (+0.1% formic acid) over 3.0 min. Chromatograms were recorded with a UV detector set at 254 nm and a time-of-flight mass spectrometer (Waters Xevo G2-XS).

1-((1*R*,5*S*)-3-(7-(8-chloronaphthalen-1-yl)-8-fluoro-2-((tetrahydro-1*H*-pyrrolizin-7a(5*H*)-yl)methoxy)pyrido[4,3-*d*]pyrimidin-4-yl)-3,8-diazabicyclo[3.2.1]octan-8-yl)butane-1,3-dione (2)

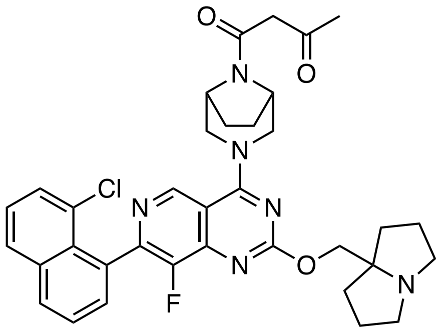

(1*R*,5*S*)-3-(7-(8-chloronaphthalen-1-yl)-8-fluoro-2-((tetrahydro-1*H*-pyrrolizin-7a(5*H*)-yl)methoxy)pyrido[4,3-*d*]pyrimidin-4-yl)-3,8-diazabicyclo[3.2.1]octan-8-ium-di-2,2,2-trifluoroacetate (50.0 mg, 0.064 mmol, 1.0 equiv) was dissolved in dry dichloroethane (1 mL). DIPEA (28.1 mg, 0.218 mmol, 3.4 equiv) and 2,2,6-trimethyl-4*H*-1,3-dioxin-4-one (12.4 mg, 0.087 mmol, 1.4 equiv) were subsequently added and the solution was stirred at 80 ºC for 1 h. The reaction mixture was partitioned between saturated aqueous sodium bicarbonate solution and dichloromethane. The layers were separated, the organic phase was extracted with dichloromethane, dried over Na_2SO_4, filtered, and concentrated. The crude mixture was purified by reverse-phase HPLC to yield **(1*R*,5*S*,8*s*)-3-(7-(8-chloronaphthalen-1-yl)-8-fluoro-2-((hexahydropyrrolizin-4-ium-7a(1*H*)-yl)methoxy)pyrido[4,3-*d*]pyrimidin-4-yl)-8-(3-oxobutanoyl)-3,8-diazabicyclo[3.2.1]octan-8-ium-di-2,2,2-trifluoroacetate** as colorless solid (27.8 mg, 0.032 mmol, 50%). ^{1}H NMR (600 MHz, d6-Acetone) δ 9.25 – 9.20 (m, 1H), 8.20 (d, *J* = 7.7 Hz, 1H), 8.09 (d, *J* = 8.0 Hz, 1H), 7.76 – 7.71 (m, 1H), 7.65 (dd, *J* = 7.2, 2.6 Hz, 2H), 7.57 (t, *J* = 7.8 Hz, 1H), 4.80 (td, *J* = 13.2, 8.3 Hz, 5H), 4.50 (d, *J* = 5.9 Hz, 1H), 3.91 – 3.69 (m, 5H), 3.40 (s, 2H), 2.43 – 2.36 (m, 2H), 2.33 – 2.27 (m, 2H), 2.26 (s, 3H), 2.24 – 2.17 (m, 4H), 1.97 – 1.81 (m, 4H). ^{19}F NMR (600 MHz, d6-Acetone) δ -140.4. HRMS: m/z calcd. for $C_{35}H_{37}ClFN_6O_3^+$ ([M+H]$^+$): 643.2594, found: 643.2711.

1-((1*R*,5*S*)-3-(7-(8-chloronaphthalen-1-yl)-8-fluoro-2-((tetrahydro-1*H*-pyrrolizin-7a(5*H*)-yl)methoxy)pyrido[4,3-*d*]pyrimidin-4-yl)-3,8-diazabicyclo[3.2.1]octan-8-yl)-2,2-dihydroxybutane-1,3-dione (3)

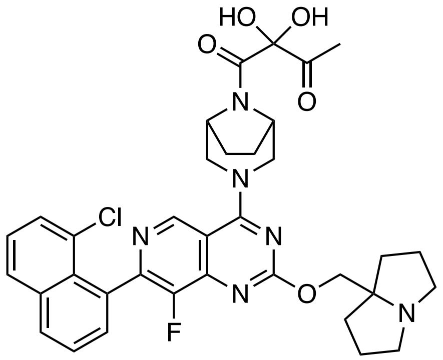

(1*R*,5*S*,8*s*)-3-(7-(8-chloronaphthalen-1-yl)-8-fluoro-2-((hexahydropyrrolizin-4-ium-7a(1*H*)-yl)methoxy)pyrido[4,3-*d*]pyrimidin-4-yl)-8-(3-oxobutanoyl)-3,8-diazabicyclo[3.2.1]octan-8-ium-di-2,2,2-trifluoroacetate (22.7 mg, 0.026 mmol, 1.0 equiv.) was dissolved in wet CH_2Cl_2 (1 mL). Pyridine (8.24 mg, 0.104 mmol, 4.0 equiv.) and dess-martin periodinane (44.2 mg, 0.104 mmol, 4.0 equiv.) were added and the mixture was stirred for 1 h at room temperature. Solvents were removed under reduced pressure. The residue was dissolved in MeCN:H2O (1:1) and purified by reverse phase HPLC (C18) to yield **1-((1*R*,5*S*)-3-(7-(8-chloronaphthalen-1-yl)-8-fluoro-2-((tetrahydro-1*H*-pyrrolizin-7a(5*H*)-yl)methoxy)pyrido[4,3-*d*]pyrimidin-4-yl)-3,8-diazabicyclo[3.2.1]octan-8-yl)-2,2-dihydroxybutane-1,3-dione-di-2,2,2-trifluoroacetate** as colorless solid (7.1 mg, 0.079 mmol, 30%). ^{1}H NMR (400 MHz, d6-Acetone) δ 9.22 – 9.17 (m, 1H), 8.20 (dd, *J* = 8.2, 1.1 Hz, 1H), 8.13 – 8.06 (m, 1H), 7.78 – 7.71 (m, 1H), 7.68 – 7.63 (m, 2H), 7.58 (t, *J* = 7.8 Hz, 1H), 4.92 – 4.68 (m, 6H), 3.90 – 3.80 (m, 4H), 3.38 (s, 2H), 2.46 – 2.39 (m, 2H), 2.32 (s, 3H), 2.23 (ddt, *J* = 25.6, 12.8, 6.8 Hz, 6H), 1.94 (t, *J* = 13.5 Hz, 4H). ^{19}F NMR (376 MHz, d6-Acetone) δ -76.2, -140.6. HRMS: m/z calcd. for $C_{35}H_{37}ClFN_6O_5^+$ ($[M+H]^+$): 675.2493, found: 675.2487.

(*S*)-2-(4-(7-(8-chloronaphthalen-1-yl)-8-fluoro-2-((tetrahydro-1*H*-pyrrolizin-7a(5*H*)-yl)methoxy)pyrido[4,3-*d*]pyrimidin-4-yl)-1-(3-oxobutanoyl)piperazin-2-yl)acetonitrile

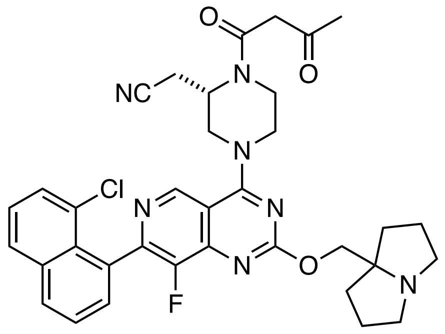

(*S*)-2-(4-(7-(8-chloronaphthalen-1-yl)-8-fluoro-2-((tetrahydro-1*H*-pyrrolizin-7a(5*H*)-yl)methoxy)pyrido[4,3-*d*]pyrimidin-4-yl)piperazin-2-yl)acetonitrile (50.0 mg, 0.073 mmol, 1.0 equiv) was dissolved in dry toluene (1 mL). DIPEA (28.3 mg, 0.219 mmol, 3 equiv) and 2,2,6-trimethyl-4*H*-1,3-dioxin-4-one (12.4 mg, 0.087 mmol, 1.2 equiv) were subsequently added and the solution was stirred at 80 ºC for 1 h. The reaction mixture was partitioned between saturated aqueous sodium bicarbonate solution and dichloromethane. The layers were separated, the organic phase was extracted with dichloromethane, dried over Na_2SO_4, filtered, and concentrated. The crude mixture was purified by reverse-phase HPLC to yield **(*S*)-2-(4-(7-(8-chloronaphthalen-1-yl)-8-fluoro-2-((tetrahydro-1*H*-pyrrolizin-7a(5*H*)-yl)methoxy)pyrido[4,3-*d*]pyrimidin-4-yl)-1-(3-oxobutanoyl)piperazin-2-yl)acetonitrile** as colorless solid (27.4 mg, 0.042 mmol, 57%). HRMS: m/z calcd. for $C_{35}H_{36}ClFN_7O_3^+$ ([M+H]$^+$): 656.2547, found: 656.2547.

(*S*)-2-(4-(7-(8-chloronaphthalen-1-yl)-8-fluoro-2-((tetrahydro-1*H*-pyrrolizin-7a(5*H*)-yl)methoxy)pyrido[4,3-*d*]pyrimidin-4-yl)-1-(2,2-dihydroxy-3-oxobutanoyl)piperazin-2-yl)acetonitrile (4)

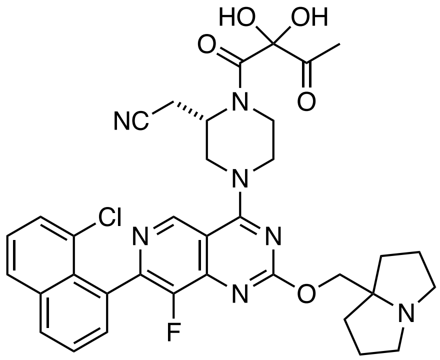

(*S*)-2-(4-(7-(8-chloronaphthalen-1-yl)-8-fluoro-2-((tetrahydro-1*H*-pyrrolizin-7a(5*H*)-yl)methoxy)pyrido[4,3-*d*]pyrimidin-4-yl)-1-(3-oxobutanoyl)piperazin-2-yl)acetonitrile (20.0 mg, 0.030 mmol, 1.0 equiv.) was dissolved in wet CH_2Cl_2 (0.2 mL). Pyridine (9.6 mg, 0.122 mmol, 4.0 equiv.) and dess-martin periodinane (51.7 mg, 0.122 mmol, 4.0 equiv.) were added and the mixture was stirred for 1 h at room temperature. Solvents were removed under reduced pressure. The residue was dissolved in MeCN:H2O (1:1) and purified by reverse phase HPLC (C18) to yield **(*S*)-2-(4-(7-(8-chloronaphthalen-1-yl)-8-fluoro-2-((tetrahydro-1*H*-pyrrolizin-7a(5*H*)-yl)methoxy)pyrido[4,3-*d*]pyrimidin-4-yl)-1-(2,2-dihydroxy-3-oxobutanoyl)piperazin-2-yl)acetonitrile (4)** as colorless solid (11.2 mg, 0.016 mmol, 53%). HRMS: m/z calcd. for $C_{35}H_{36}ClFN_7O_5^+$ ($[M+H]^+$): 688.2445, found: 688.2444.

NMR Spectra

1-((1*R*,5*S*)-3-(7-(8-chloronaphthalen-1-yl)-8-fluoro-2-((tetrahydro-1*H*-pyrrolizin-7a(5*H*)-yl)methoxy)pyrido[4,3-*d*]pyrimidin-4-yl)-3,8-diazabicyclo[3.2.1]octan-8-yl)butane-1,3-dione (**2**)

^{1}H NMR

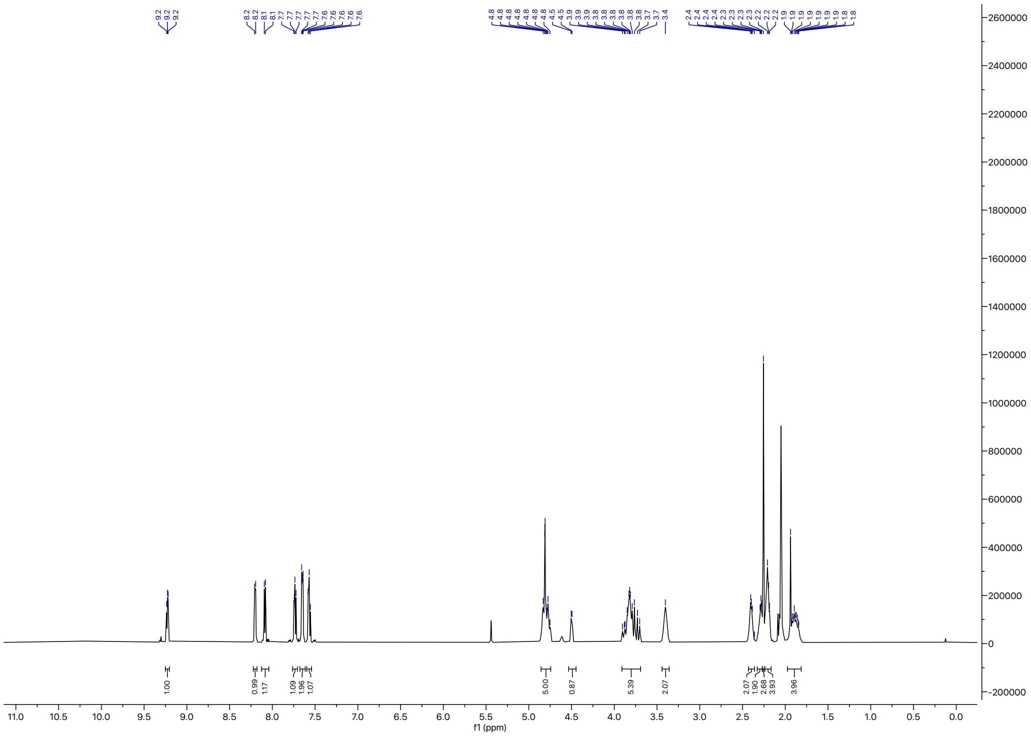

^{19}F NMR

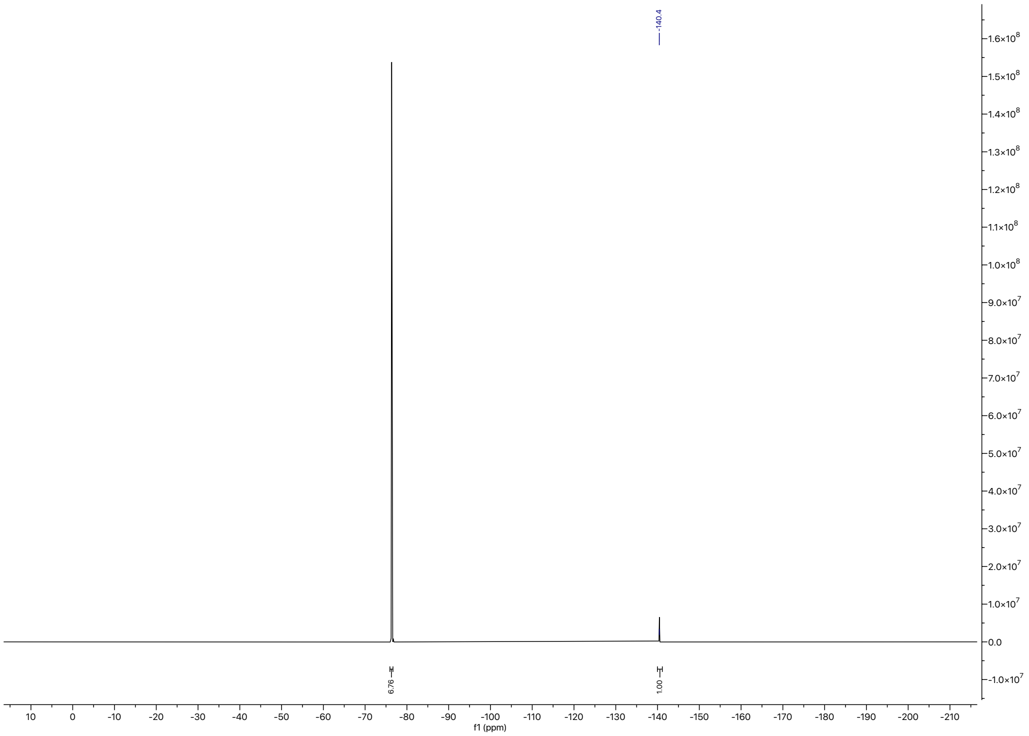

1-((1*R*,5*S*)-3-(7-(8-chloronaphthalen-1-yl)-8-fluoro-2-((tetrahydro-1*H*-pyrrolizin-7a(5*H*)-yl)methoxy)pyrido[4,3-*d*]pyrimidin-4-yl)-3,8-diazabicyclo[3.2.1]octan-8-yl)-2,2-dihydroxybutane-1,3-dione (**3**)

^{1}H NMR

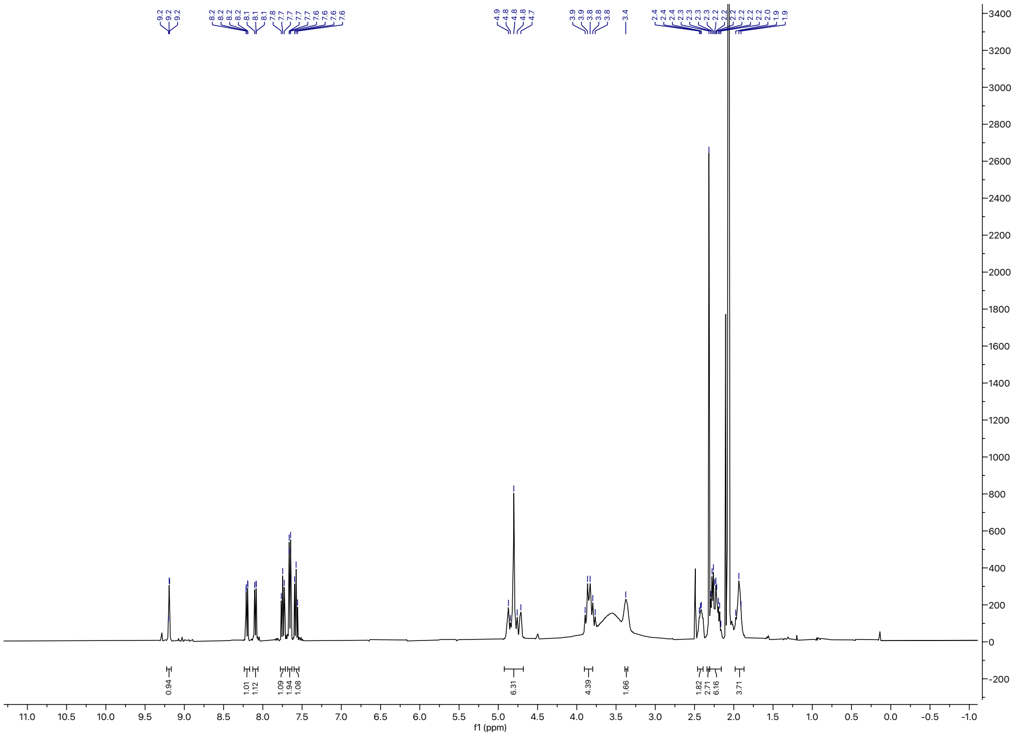

^{19}F NMR

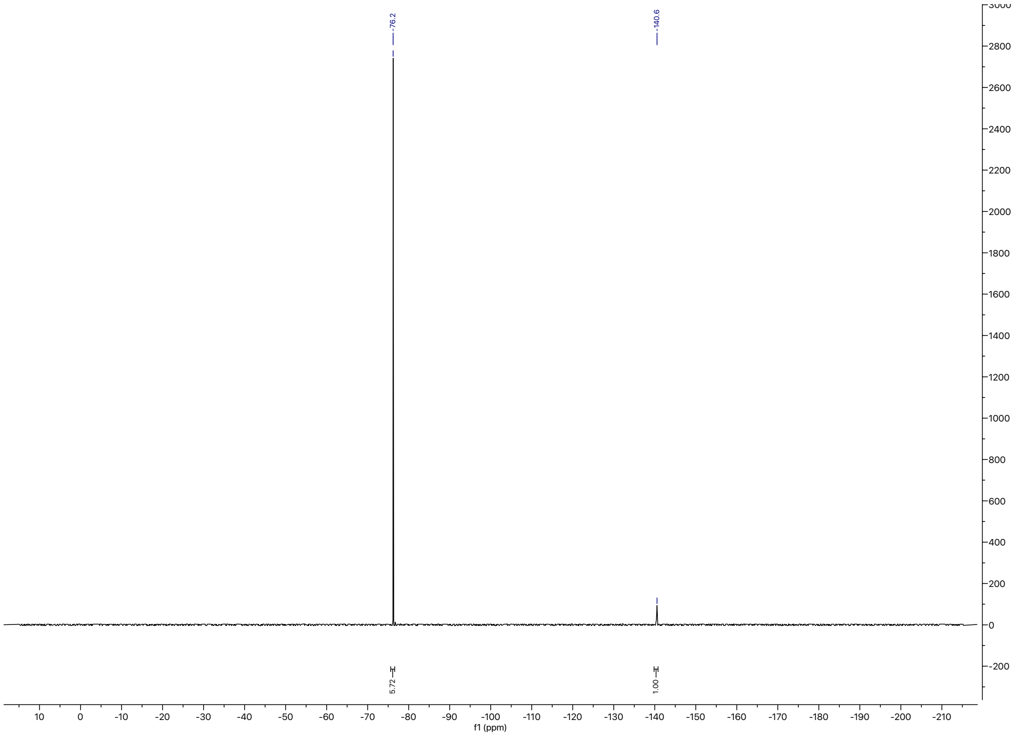

References for Supporting Information

(1) Ostrem, J. M.; Peters, U.; Sos, M. L.; Wells, J. A.; Shokat, K. M. K-Ras(G12C) Inhibitors Allosterically Control GTP Affinity and Effector Interactions. *Nature* 2013, *503* (7477), 548–551.

(2) Ahmadian, M. R.; Zor, T.; Vogt, D.; Kabsch, W.; Selinger, Z.; Wittinghofer, a; Scheffzek, K. Guanosine Triphosphatase Stimulation of Oncogenic Ras Mutants. *Proc. Natl. Acad. Sci. U. S. A.* 1999, *96* (12), 7065–7070.

(3) Huehls, A. M.; Coupet, T. A.; Sentman, C. L. Bispecific T-Cell Engagers for Cancer Immunotherapy. *Immunol. Cell Biol.* 2014, *93* (10), 290–296.

(4) Maurer, T.; Garrenton, L. S.; Oh, A.; Pitts, K.; Anderson, D. J.; Skelton, N. J.; Fauber, B. P.; Pan, B.; Malek, S.; Stokoe, D.; Ludlam, M. J. C.; Bowman, K. K.; Wu, J.; Giannetti, A. M.; Starovasnik, M. A.; Mellman, I.; Jackson, P. K.; Rudolph, J.; Wang, W.; Fang, G. Small-Molecule Ligands Bind to a Distinct Pocket in Ras and Inhibit SOS-Mediated Nucleotide Exchange Activity. *Proc. Natl. Acad. Sci. U. S. A.* 2012, *109* (14), 5299–5304.

(5) Gibson, D. G.; Young, L.; Chuang, R. Y.; Venter, J. C.; Hutchison, C. A.; Smith, H. O. Enzymatic Assembly of DNA Molecules up to Several Hundred Kilobases. *Nat. Methods* 2009, *6* (5), 343–345.

(6) Gentile, D. R.; Rathinaswamy, M. K.; Jenkins, M. L.; Moss, S. M.; Siempelkamp, B. D.; Renslo, A. R.; Burke, J. E.; Shokat, K. M. Ras Binder Induces a Modified Switch-II Pocket in GTP and GDP States. *Cell Chem. Biol.* 2017, *24* (12), 1455-1466.e14.

(7) Sondermann, H.; Soisson, S. M.; Boykevisch, S.; Yang, S. S.; Bar-Sagi, D.; Kuriyan, J. Structural Analysis of Autoinhibition in the Ras Activator Son of Sevenless. *Cell* 2004, *119* (3), 393–405.
